# Cinnamon: A Natural Feed Additive for Poultry Health and Production—A Review

**DOI:** 10.3390/ani11072026

**Published:** 2021-07-07

**Authors:** Akhtar Ali, Eric N. Ponnampalam, Gamini Pushpakumara, Jeremy J. Cottrell, Hafiz A. R. Suleria, Frank R. Dunshea

**Affiliations:** 1 Faculty of Veterinary and Agricultural Sciences, School of Agriculture and Food, The University of Melbourne, Parkville, VIC 3010, Australia; akali@student.unimelb.edu.au (A.A.); jcottrell@unimelb.edu.au (J.J.C.); hafiz.suleria@unimelb.edu.au (H.A.R.S.); 2 Animal Production Sciences, Agriculture Victoria Research, Department of Jobs, Precincts and Regions, Bundoora, VIC 3083, Australia; Eric.Ponnampalam@agriculture.vic.gov.au; 3 Faculty of Agriculture, University of Peradeniya, Peradeniya 20400, Sri Lanka; ngpkumara@pdn.ac.lk; 4 Faculty of Biological Sciences, University of Leeds, Leeds LS2 9JT, UK

**Keywords:** cinnamon, natural feed additive, gut microbiota, poultry health, immune response

## Abstract

**Simple Summary:**

Due to restrictions on antibiotics in chicken production in recent years, the poultry industry has looked towards alternatives such as plant-derived feed additives. Plant bioactive compounds, such as phytochemicals, in poultry diets, are gaining popularity due to their potential antioxidant and anti-microbial activities. Some plant derivatives improve the immune system, reduce the stress response, and exert positive effects on health and performance. The dietary supplementation of cinnamon in poultry feed as a natural feed additive has beneficial impacts on nutrient digestibility, hypocholesterolaemic, blood biochemical profile, gene expression, immunity, and particularly on gut health to alleviate the impact of disease and heat stress by maintaining water and electrolytic balance and feed intake. It is clearly demonstrated that cinnamon can be used as an alternative to antibiotics in the poultry industry offering greater animal health, food safety, and economic aspects of poultry production.

**Abstract:**

The increased bacterial resistance to synthetic antibiotics and consumer awareness about the health and food safety concerns have triggered the ban on the use of antibiotic growth promotors (AGPs) in the poultry industry. This situation encouraged the poultry sector and industry to explore safe alternatives to AGPs and focus on developing more sustainable feed management strategies to improve the intestinal health and growth performance of poultry. Consequently, phytogenic feed additives (PFAs) have emerged as natural alternatives to AGPs and have great potential in the poultry industry. In recent years, cinnamon (one of the most widely used spices) has attracted attention from researchers as a natural product with numerous health benefits for poultry. The essential oils in cinnamon, in particular, are of interest because of their antioxidant, anti-microbial, anti-inflammatory, antifungal, and hypocholesterolaemic effects, in addition to their ability to stimulate digestive enzymes in the gut. This review mainly emphasizes the potential impact of cinnamon as a natural feed additive on overall gut health, nutrient digestibility, blood biochemical profile, gene expression, gut microbiota and immune response.

## 1. Introduction

The demand for poultry meat and meat products has increased in recent years. The global production of chicken meat reached about 137 million tons in 2020, making poultry the most consumed meat in the world. Therefore, the poultry industry contributes significantly to animal protein consumption and to human nutrition and global food security [1]. In recent years, significant improvements have been made to improve poultry health and performance. Feed is the major component in the poultry industry, exposing the gastrointestinal tract to a wide range of factors that may affect gut health. The gastrointestinal tract is considered a highly complex and dynamic organ that plays a pivotal role in gut health. Several stressors may negatively impact the balance in the gut ecosystem and, ultimately health status and productivity of poultry. Stressors such as heat stress and gastrointestinal dysbiosis are considered major threats to the poultry industry through their impacts on gut health and increased disease susceptibility. The gut from healthy animals can efficiently digest and absorb the nutrients under environments where these stressors are minimized [2].

Previously, antibiotic growth promoters were used to control gastrointestinal pathogens and reduce the effects of stressors on gut function. However, growing consumer awareness about the adverse effects of antibiotics on human health coupled with increased bacterial resistance and concerns about food safety have led to the imposition of restrictions on the use of antibiotics in poultry production. This situation has resulted in researchers and industry exploring alternatives to AGPs with their attention focused on developing more sustainable dietary interventions to improve the gut microbiome and overall health of poultry. PFAs have emerged as alternatives to AGPs and have great potential in the poultry industry [3]. For example, under conditions of heat stress in poultry they have been shown to improve the immune system, exert positive effects on health performance, and reduce stress response [1,4,5].

**Figure 1:**
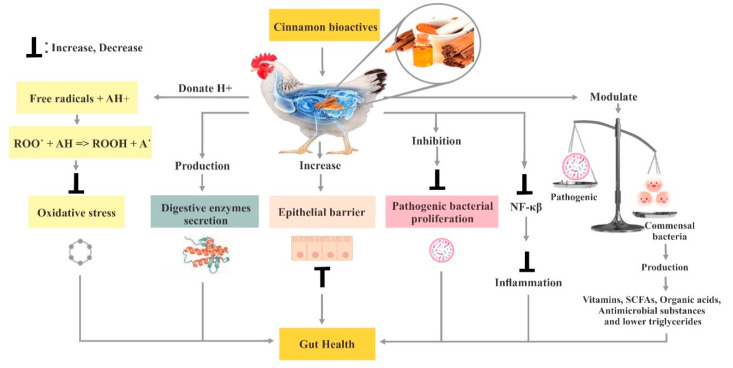
Effect of cinnamon on poultry gut health.

Furthermore, consumers are demanding poultry meat with less fat, and the search for safe growth promotants and carcass modifiers has also become a priority research area. Thus, PFAs have been studied as alternatives to AGPs and growth modifiers to provide safe and wholesome food. Natural antioxidants found in many PFAs have been found effective in extending the shelf life, meat quality and acceptability of poultry meat. Phytochemicals from herbs and spices have attracted particular attention as alternatives to AGPs due to their beneficial properties. Being natural, non-toxic, chemical residue-free and easy availability made them highly acceptable in the poultry industry. Natural products from plants have been found to have beneficial effects viz. appetizer, increased digestive enzymes secretion, immuno-stimulant, bactericidal, antiviral and antioxidants in animals. Cinnamon is one of the most potent PFAs, which has great potential for poultry. Therefore, there is a crucial need to review the impacts of cinnamon bioactive compounds on the gut microflora and overall health performance of chicken and develop various effective mitigation strategies to overcome the significant production loss and attain successful production in hot climatic regions or areas. Overall, the effect of cinnamon on poultry gut health is given in Figure 1.

## 2. Cinnamon

Cinnamon belongs to the genus *Cinnamomum* (Lauraceae family) which contains more than 250–300 aromatic evergreen shrubs and plant trees [6,7,8]. However, only a few of these species have significant economic importance worldwide as a common spice including *Cinnamomum zeylanicum* (*C. zeylanicum*: True Sri Lankan cinnamon), *C. cassia* (Chinese cinnamon), *C. burmanni* (*Indonesian cinnamon*) and *C. loureiori* (*Vietnamese cinnamon*). The annual production of cinnamon is around 0.23 million metric tons, mainly cultivated in Indonesia, Sri Lanka, China, India, Vietnam, and Madagascar.

### Phytochemistry of Cinnamon

Phytochemicals are plant bioactive non-nutritive compounds that are usually found in small quantities [9]. They have different classes according to their structure and include phenolic compounds, phytosterols, phytoestrogens, glucosinolates, saponins, terpenoids, protease inhibitors and organo-sulfur containing compounds [10]. They have significant antioxidant capacity to reduce and protect oxidative stress [11]. Cinnamon consists of various bioactive compounds. Modern analytical techniques have enabled the characterization, identification, purification and quantification of individual compounds and the study of their potent biological activities [12]. Generally, gas chromatography is applied to characterize volatile compounds while liquid chromatography for the identification of phenolic compounds [13]. It is documented that cinnamon consists of natural antioxidant, anti-microbial and anti-inflammatory components such as volatile oils, flavonoids, curcuminoids, coumarins, tannins, alkaloids, xanthones, terpenoids, phenolics and other compounds in significant amounts [14,15]. The concentration of volatile compounds in cinnamon essential oil (CNO) mainly depends upon the plant parts (leaves, bark, root, stem) from which it is extracted. About forty-one volatile compounds were identified from the bark oil of cinnamon (*C. cassia*) tree [16]. Cinnamaldehyde (55% to 78%) is the main flavor compound in CNO extracted from bark while eugenol (59–78%) is the main compound in CNO that is extracted from leaves [17]. The volatile oil is approximately 0.6–1% and 1–2% phlobatannins, calcium oxalate, starch, mucilage, and mannitol (sweet) in the bark. Moreover, Kim, et al. [18] further investigated the cinnamon bark oil through GC-MS (gas chromatography–mass spectrometry) and identified seventeen different bioactive compounds. The major bioactive compounds of cinnamon are cinnamaldehyde, cinnamate, cinnamic acid, all of which play vital roles in various biological activities [19,20]. The different essential oils that have been reported in cinnamon include trans-cinnamaldehyde, eugenol, cinnamyl acetate, L-borneol, L-bornyl acetate, β-caryophyllene, caryophyllene oxide, E-nerolidol, α-thujene, α-cubebene, terpinolene and α-terpine [19]. The LC-MS (liquid chromatography–mass spectrometry) analysis has shown that the concentrations of condensed tannins, proanthocyanidins (PAs) and epicatechin in cinnamon are 26.8%, 23.2% and 3.6%, respectively. Cinnamon has a high polyphenol contents [21] and the anthocyanidins (A and B procyanidins) are also present in cinnamon [12].

## 3. Poultry Gut Health

Efficient immune system development and proper digestion and absorption of feed, water, and electrolyte balance in the gut leads to the development of strong gut health in poultry. The gut ecosystem plays a vital role in eliminating toxins and infectious agents from the intestinal tract of the poultry. Many factors influence the gut microbial ecosystem, including feed additives (phytobiotics, prebiotics, probiotics, feed enzymes, organic acids etc.), feed composition, genetics, heat stress, feeding practices on the poultry farm, among others. These factors exert a substantial impact on the gut microbiota and poultry health [22]. The association between gut health and poultry performance is widely accepted with optimal health including proper physiological functions of the intestinal tract, morphological integrity, efficient immune response, developed barrier functions, energy balance, tissue metabolism, sustained inflammatory balance and sufficient microbiota to perform desired functions in the gut. The health of poultry is influenced by the structure and functionality of gut microbiota. The progression of acquisition and maturation of the intestinal microbiota throughout the growth period of the poultry has a marked impact on the modulation of physiological functions (nutrient digestion, immunity, intestinal barrier integrity etc.) to maintain gut homeostasis and development of the intestinal epithelium. These functions are essential to optimize energy use and efficiency of extraction by the poultry birds [2].

The intestinal microbiota of poultry birds is a composite community of diverse microorganisms. The intestinal microbiota of chicken usually is dominated at the phylum level with hundreds of Actinobacteria, Bacteroidetes, Firmicutes, Fusobacteria and Proteobacteria species. The poultry gastrointestinal tract comprises various sections (crop, gizzard, ileum, cecum and colon) with specific environments and physiological roles that initiate a spatial supply of complex microbial ecosystem. The gastrointestinal tract (GIT) contains 500–1000 various bacteria species and 100 trillion cells [23]. The poultry gut ecosystem consists of bacteria, protozoa and fungi in various proportions. The microbiota concentration varies throughout the intestinal tract, which is maximum at distal segments in poultry birds. The intestinal epithelial has tight junctions between the cells to prevent the invasion of the microorganism and participates in cellular signaling. Many studies have suggested that the interactions between the mucosa and pathogenic microbes or their toxins trigger oxidative stress, which leads to the destruction of tight junctions and the intestinal epithelial barrier, intestinal mucosa and lipid peroxidation. As a result, the infected poultry birds exhibit decreased feed intake, digestion, and nutrient absorption in the gut result in lower growth performance. The inclusion of dietary antioxidant compounds as feed additives helps to reduce free radicals and maintains the intestinal mucosa.

Consequently, it is pivotal to articulate a cost-effective approach to mitigating oxidative stress in the poultry industry. The supplementation of bioactive compounds in poultry feed improves the antioxidant ability, immunity and health performance. Cinnamon contains very active compounds, including essential oils (EOs) and phenolics, which possess potent anti-microbial, anti-inflammatory, and antioxidant activities that act as defensive agents against oxidative damage in the chicken intestinal tract.

### 3.1. Utilization of Cinnamon in Poultry Feed

Cinnamon is one of the PFAs that have been approved for inclusion in poultry feed by the US Food and Drug Administration (FDA). Since 2000, bioactive compounds including EOs, cinnamaldehyde, phenolic compounds and others have been included in poultry feed to improve immunity, metabolism, health, growth performance, carcass traits and meat quality. The bioactive compounds of cinnamon have potent anti-inflammatory, anti-microbial and antioxidant properties with free radical scavenging actions and strong inhibitory effects on nitric oxide (NO) production by inhibiting the activity of NFκβ [24]. In studies conducted with broiler chickens, anti-microbial (antiviral, antibacterial and antifungal) activities against many microorganisms and strong antioxidant activities have been observed in poultry diets supplemented with cinnamon and their EOs. Furthermore, CNO has potent antioxidant, hypocholesterolaemic, anticandidal and analgesic activities [25]. Concomitantly, cinnamon bioactive compounds can disrupt the growth of pathogenic microbes and stimulate the growth of commensal bacteria in the intestinal tract of poultry birds [26].

### 3.2. Impact of Cinnamon on the Digestibility of Nutrients

Improved utilization of feed improves the feed conversion ratio (FCR), body weight gain (BWG) and overall health performance of broiler chicken. The stabilization of the gut microbiota ecosystem and the stimulation of digestive enzymes secretion are the two well-accepted mechanisms that play a leading role in improving feed utilization and inhibiting the growth-depressing ailments related to metabolism and digestion [27,28,29]. The potential impacts of CNO on the secretion of digestive enzymes from the intestinal mucosa and pancreas have been described in many poultry studies [30,31,32]. These positive impacts had been confirmed to improve the digestibility of nutrients [31,33]. Additionally, the bioactive compounds of cinnamon affect lipid metabolism by transporting the fatty acids in the digestive tract of broilers. The CNO has positive effects on the secretion of digestive enzymes and improves the digestibility of nutrients in the gut [34,35].

The CNO may also improve nutrient uptake by protecting intestinal gut morphology and integrity. For example, Devi, et al. [36] reported that digestibility of nutrients was better in the cinnamon fed group. Supplementation of CNO in broilers diet increased the villus height (VH) in the duodenum and jejunum with associated increased villus surface area and the efficiency of absorption and digestion of nutrients. In addition, a greater VH means greater mucosal digestive enzyme activity, which ultimately improves the digestibility of nutrients [37]. The increased VH was attributed to the antioxidant activity of CNO [38]. In addition, the digestive process liberates reactive oxygen species (ROS) which act on intestinal mucosa and shorten the intestinal villi, but antioxidant enzymes (catalase and superoxide) bind the ROS. The CNO acts as hydrogen donor and exhibits antioxidant activity which protect the intestinal villi from oxidative damage by stimulating the activity of these antioxidant enzymes [38]. CNO helps to decrease the pathogenic bacteria in the gut, which improves intestinal morphology [39].

Cinnamaldehyde is considered a digestion stimulating agent which enhances the digestive system of broiler chicken. For example, cinnamaldehyde increased the activity of pancreatic and intestinal enzymes by provoking the secretion of salivary glands, which ultimately improved the digestion of broiler chickens [40]. Recent studies suggested that tannins (water-soluble phenolic compounds) from cinnamon significantly impact augmenting poultry health and nutrition as they can precipitate proteins in the gut [41,42]. The apparent ileal digestibility of nutrients, crude fat and amino acids (histidine, lysine, serine, phenylalanine, asparagine and threonine) digestibility were significant in cinnamaldehyde supplemented groups [43]. Moreover, the inclusion of CNO in broiler diets improved protein digestion via increasing the secretion of hydrochloric acid (HCl) and pepsin in the gut. The CNO has a positive impact on the poultry digestive system by restoring the balance of the gut ecosystem and improving nutrient absorption, which could be attributed to the terpenoid compounds of cinnamon [44]. Phytobiotic growth promoters remained active throughout the gastrointestinal tract to exert broad-spectrum anti-microbial action, enhanced nutrient utilization, and augmented intestinal histomorphology and enhanced host immunity. The CNO stimulates the secretion of enzymes and bile acid, which improves the apparent digestibility of nutrients and fat [45,46]. The CNO play a crucial role in the interactions of feed and enzymes in the gut and influence the transit of digesta in the gut [34]. The exact mechanism by which CNO improves nutrient digestibility in the gut of poultry is unknown and needs to be elucidated.

### 3.3. Cinnamon and Blood Biochemical Profile

It is established that hematological values are dependent on the physiological state of the birds. The blood biochemical profile is a vital tool that has been used to screen the impacts of nutritional, therapeutic and environmental interventions in veterinary and human medicine. Many studies have been conducted to illustrate the effect of cinnamon on blood biochemical profile, including antioxidant activity, lipid profile and immunity. The cinnamon-based PFAs group exhibited significant differences in albumin/globulin (A/G) ratio and cholesterol concentrations as compared to control treatments [1]. Additionally, non-significant interactions were found for total protein, globulin levels and serum albumin among treatments. It was found that cinnamon-based diets played a vital role in reducing cholesterol concentrations which is highly desirable for consumers. However, it should be noted that the effects of dietary cinnamon trials on blood chemical profiles and immune system responses in poultry have not been consistent. A possible source of this variation may be related to health status of the birds. Kettunen, et al. [47] reported that CNO supplemented diets improved the immunoglobulin A concentrations and intestinal immunocompetence which ultimately increased the performance of chicks. Recently published reports have shown that cinnamaldehyde, cinnamon powder and CNO alone or in combinations with other feed additives have a wide array of potential impacts on blood chemical profile of poultry birds.

A study conducted by Al-Kassie [48] showed that broilers fed on 200 ppm dietary CNO had significantly lower cholesterol concentrations and heterophils/lymphocytes (H/L) ratio. In contrast, total protein, hemoglobin, red and white blood cells concentrations and packed cell volume were all increased. Moreover, Ciftci, et al. [49] reported that serum levels of glutathione peroxidase, catalase enzyme activities, total unsaturated fatty acids, ω-6 fatty acids and phagocytic activity of blood were significantly increased in chicks fed on diets supplemented with 1000 ppm CNO. In addition, cholesterol and malondialdehyde (MDA) concentrations, alanine aminotransferase activity and total saturated fatty acid ratio were reduced in broilers fed on CNO. These authors concluded that CNO had strong antioxidant potential. Almost similar results were found by Yang, et al. [4] who reported that MDA concentrations decreased on 21 day while serum immune globulin M (IgM) contents increased on 42 days in broilers. In another study, Mehdipour and Afsharmanesh [50] found that dietary cinnamon powder significantly increased total superoxide dismutase activity, total antioxidant capacity, corticosteroid and catalase on day 42 of the study. The concentration of MDA was reduced in the cinnamon fed group. Kanani, et al. [51] investigated the impact of cinnamon powder on blood parameters of broiler chicks (Ross 300) under heat stress. They reported that the concentration of MDA, lactate dehydrogenase and blood uric were decreased. While, there were no effect on blood sodium, urea, chlorine, potassium, creatinine and aspartate aminotransferase among all treatments. Naderi, et al. [52] found that inclusion of cinnamon and turmeric in broilers diet lowered the heterophil to lymphocyte ratio leading them to suggest that dietary cinnamon and turmeric can be used as potential stress alleviators and alternatives to AGPs. Furthermore, Toghyani, et al. [53] found that serum glutamic pyruvic transaminase (SGPT) enzyme was reduced but serum glutamic oxaloacetic transaminase (SGOT), serum total protein, albumin and triglycerides were not changed among the treatments. These outcomes are due to the antioxidant potential of cinnamaldehyde which helps to protect the liver from reactive oxygen species.

Broiler chicks supplemented diet with CNO had significantly decreased circulating cholesterol, low-density lipoprotein (LDL) and triglyceride concentrations, whereas high-density lipoproteins (HDL) were increased [54]. Similar observations were made by Najafi and Taherpour [55] who showed that 0.8% dietary cinnamon reduced plasma cholesterol, LDL, total proteins and blood glucose concentrations. In another study, Hossain, et al. [56] found that 1.0% cinnamon powder increased the antibody SP ratio (ratio of sample to positive control) for Newcastle disease and lowered blood glucose concentrations. Furthermore, dietary cinnamon and zinc decreased blood glucose and triglyceride concentrations under cold stress conditions [57]. However, there was no effects of diet on plasma cholesterol, albumin and uric acid concentrations. In another study, thiobarbituric acid, LDL and glucose concentrations were decreased in pigs supplemented with cinnamon although plasma cholesterol and triglycerides concentrations were unchanged [58].

Moreover, inclusion of dietary cinnamon significantly increased serum immunoglobulin levels in broilers [59]. Furthermore, poultry fed a diet contaminated with *Clostridium perfringens* and supplemented with cinnamaldehyde, thyme, anise, yucca extract, carvacrol and oregano essential oils (OEOs) exhibited no significant effect of diet on circulating total protein, albumin and glucose concentrations and aspartate aminotransferase activity [60]. Lee, et al. [29] found that inclusion of cinnamaldehyde in broiler diet had no significant impact on plasma lipid concentrations. In summary, the consensus is that the reduction of peroxidation and free radicals’ formation by cinnamon bioactives’ action ultimately diminishes oxidative stress in broilers.

### 3.4. Cinnamon and Gene Expression in Poultry

The profiling of gene expression is one of the advanced tools to elucidate the mechanisms of complex traits such as residual feed intake (RFI) in poultry [61,62] and other species. The next-generation sequencing and RNA sequencing technology have been widely used to categorize the genes and their pathways associated with complex traits in poultry which helps understand the molecular genetic mechanisms. For example, Izadnia, Tahmoorespur, Bakhtiarizadeh, Nassiri and Esmaeilkhanien [61] reported that 121 and 279 unknown genes were identified through differential gene expression for up and down regulations in chickens, respectively related to RFI and growth rate.

The quantitative trait loci (QTL) enrichment analysis identified 63 down-regulated genes related to feed efficiency traits enriched in the QTL regions [61]. The inclusion of dietary cinnamon extracts (100–200 mg) caused a significant reduction in tumor necrosis factor-alpha (TNF-α) and nuclear factor-kappa beta (NF-κβ) expression levels as compared to the group only injected with *E. coli* [63]. Moreover, Alves-Santos, et al. [64] stated that catechins could prevent the increase of NF-κβ.

### 3.5. Effect of Cinnamon on the Gut Microbiota

It is likely that the desired optimal health and performance of poultry can be achieved via modulating the intestinal microbiota and their functions with suitable dietary strategies [65]. So, PFAs are considered potential agents to improve chicken health by establishing a balanced gut ecosystem. PFAs are considered as quintessential feed additives in the poultry industry since there are no residue or toxin issues. Among the PFAs, phytobiotics are used to modulate the poultry gut ecosystem via generating anti-microbial, anti-inflammatory, and antioxidant responses, increasing the optimum nutrient absorption in the gut system [66]. To meet the market demand for poultry meat, broilers are reared to reach their maximum weight in the shortest possible time. Therefore, gut microbiota plays a crucial role to maintain the productive interactions between the host and the gut. In the intestinal ecosystem, the digestion and absorption of many nutrients takes place in the small intestine while the cecum is the site which is densely populated with a range of bacteria which are primary responsible for the fermentation of nutrients not absorbed at the ileum [67]. The chicken ileum is predominant with microaerophilic bacteria (*Enterococcus* spp. and *Lactobacillus* spp.) while the cecum nurtures the pathogenic obligate anaerobic bacteria (*Campylobacter* spp. and *Enterococcus* spp.) [68,69]. The intestinal ecosystem has been proposed as being closely related to poultry performance. However, very few studies have investigated the relationship between phytobiotic dietary modulation, intestinal bacteria and health and performance of poultry. Nevertheless, the next section will discuss a possible strategic approach to improve health performance through modulating the gut ecosystem and relevant gut bacteria.

The inclusion of dietary cinnamon bioactive compounds contributes to producing and maintaining gut microflora and digestive functions in poultry [35]. The dietary cinnamon promoted the growth of beneficial bacteria while reducing the pathogenic bacterial load as compared to the control groups [1]. They found that dietary cinnamon increased the growth of *Lactobacillus* spp. while inhibiting *Campylobacter* spp. and *E. coli* in the ileum and cecum of poultry. The production of SCFAs are attributed towards the *Lactobacillus* spp. fermentation which are responsible for the maintenance of gut ecosystem and preventing the pH-sensitive pathogenic bacteria. The bioactive compounds of cinnamon have potential anti-microbial activities against *Enterococcus faecalis*, *Vibrio parahaemolyticus*, *Pseudomonas aeruginosa*, *Salmonella* spp. *Klebsiella pneumoniae*, *Staphylococcus epidermis*, *Staphylococcus aureus* and *E. coli* [70]. For example, the CNO inhibited the action of Gram-negative bacteria; *Enterobacter cloacae*, *E. coli*, *Pseudomonas aeruginosa*, and *Alcaligens faecalis* and Gram-positive bacteria; *Micrococcus leteus*, *Staphylococcus aureus*, *Enterococcus faecalis*, and *Bacillus cereus*, the fungi; *Rhizopus oligosporus* and *Aspergillus niger*, and the yeast; *Candida albicans* [71].

The proportions of commensal and pathogenic bacteria in the poultry gut system should be balanced to achieve optimal animal health and productivity. The potential anti-microbial and antioxidant effects had been reported in many studies [4,50]. The CNOs, including cinnamaldehyde, carvacrol, thymol, etc. showed powerful antioxidant and anti-microbial activities against *Salmonella* and *E. coli* [72]. The inclusion of CNO in broilers diet had significantly increased *Lactobacillus* and *Bifidobacteria* in the cecum while reduced the *E. coli* relative multiplicity on 42 day reported by Yang, Zhao, Shao, Liao, Zhang, Lu and Luo [4]. They suggested that dietary CNO can be used as an effective alternative to AGPs to improve gut microbiota in the cecum of broilers. Similar results were observed by [50], who found that the inclusion of 200 ppm/kg dietary CNO increased the numbers of *Lactobacillus* in the ileum while decreasing the *coliforms* as compared to the other groups. The CNO exhibited more potent anti-microbial results as compared to the extract against bacteria and fungi where the lowest minimum inhibitory concentration (MIC) (1.25% *v/v*) was reported against *E. coli*, *Klebsiella* spp., *Bacillus* spp. and *Listeria monocytogenes* as well as *Rhizomucor* spp. among the fungi [73]. Concomitantly, they concluded that MIC and minimal bactericide concentration for CNO were between 25–100 and 125–250 ug/mL, respectively.

Moreover, Pathak, et al. [74] reported that supplementation of cinnamaldehyde and formic acid in broilers diet did not exert any effect against total bacterial counts of *E. coli* and *Lactobacillus.* However, *Clostridium* and *Salmonella* counts were reduced in the ileal and caecal contents as compared to the other groups. Additionally, cinnamaldehyde and formic acid were more effective against *Clostridium* genus in the small intestine and caecum of broilers.

The counts of *E. coli* and *Clostridium* spp. were lower in the pre-caecal contents of broilers supplemented with CNO while *Lactobacillus* spp. were unchanged [37]. The proliferation of pathogens in the gut can be prevented by using cinnamaldehyde and thymol in poultry diet [75]. These authors reported that a blend of cinnamaldehyde and thymol reduced the *E. coli* counts in the caecum, possibly due to the capacity of CNO to disrupt the bacterial cell membranes. Additionally, the CNO stimulates mucus release in the intestinal tract, which in turn reduces the adhesion of pathogens to the epithelial cells in the gut [31]. Bacterial counts increase with age in the small intestine with the predominant species being *Streptococci*, *Lactobacilli*, *Enterobacteria*, *Eubacteria* and *Fusobacteria*. On the other hand, the gram-positive *Bacteroides, Cocci*, *Eubacterium* spp., *Lactobacillus* spp., *Clostridium* spp., and *Fusobacterium* spp. predominate in the anaerobic caecum of broilers [76].

Furthermore, Jamroz, et al. [77] reported that broilers supplemented with cinnamaldehyde, carvacrol and capsaicin had higher *Lactobacillus* counts in the gut while fungi, *Clostridium perfringens* and *E. coli* counts were reduced. The use of trans-cinnamaldehyde in in-vitro fermentations reduced Clostridium jejuni and Campylobacter concentrations after 8 h of incubation [78] without exerting any detrimental effect on natural gut microflora. Additionally, CNO has greater potency against *E. coli* and *Salmonella typhimurium* than the other EOs [79]. The largest and most abundant bioactive compounds in cinnamon are the volatile compounds: cinnamaldehyde, eugenol, carvacrol, condensed tannins and other polyphenolic compounds perform antioxidant, anti-microbial and anti-inflammatory activities in the poultry gut. The anti-microbial potential of cinnamaldehyde, carvacrol and eugenol were detected due to their preemptive effects against harmful microorganisms [80]. Furthermore, carvacrol can manipulate the pH equilibrium of inorganic ions by disrupting the membrane integrity of the gut [81]. On the other hand, herb and spice extracts can stimulate the growth of beneficial bacteria while inhibiting the growth of pathogenic bacteria in the intestinal tract of the broilers [82].

Many plant bioactive compounds are used as feed additives to improve poultry health and growth performance. These additives modulate the gut microflora of broilers to enhance nutrient availability and uptake in the gut, immune health, and inhibition of pathogenic microorganisms. The antibacterial and antifungal potential of *C. bejolghota* bark oil was firstly examined against Gram positive, Gram negative bacteria and *Colletotrichum* spp. fungi. The MIC of CNO against fungal pathogens was 125–500 μg/mL while it was 31.25 to 62.50 μL/mL against bacteria. The potent anti-microbial activity of *C. bejolghota* EO was associated mainly with compounds such as terpinen-4-ol, 1,8-cineole, borneol, linalool, and γ-terpineol [83]. Orengo, et al. [84] reported that inclusion of cinnamaldehyde in broilers diet reduced *Eimeria aceryulina* oocyst infection and decreased gross lesion scores compared with other groups. It is believed that CNOs may help reduce stress during critical conditions and stimulate a strong immune defense system, which results in improved gut health and better growth [38]. Dietary CNO supplementation has the potential to reduce the rate of *Eimeria oocyst* shedding and act as a therapeutic anticoccidial agent in poultry [85].

Cinnamon is abundant with flavonols [12,15,86,87] which have strong antioxidant and anti-microbial properties. Among flavonols, quercetin was reported to inhibit bacteria growth, such as *Salmonella enterica* serotype *Typhimurium*, *E. coli*, *Staphylococcus aureus*, and *Pseudomonas aeruginosa* in the poultry gut. Thus, the quercetin compounds enhance performance and health by modulating the gut ecosystem of poultry birds [23]. Cinnamon anthocyanins have potential effects on poultry health as they act as phyto-pigments in plants. They have potential as anti-inflammatory, antidiabetic, anticancer biochemical agents, anti-obesity, immunomodulatory and antioxidant agents; thus, inclusion of anthocyanins produce various health benefits in the gut of heat stressed broilers [5]. Proanthocyanidins with a degree of polymerization less than three are depolymerized into catechin and epicatechin mixtures of monomers and dimers absorbed from the small intestine.

Additionally, the PAs with more than 10 degrees of polymerization are not absorbed from the small intestine and pass into the large intestine, where they are degraded by the microflora [88]. Although PAs exhibit low absorption from the small intestine, they can still deliver health benefits [89]. Proanthocyanidins such as procyanidin A and procyanidin B2 are metabolized by gut flora to produce phenolic acids and other metabolites and are detected in urine. It is suggested that these phenolic acids may have health effects in the gut [90]. A variety of gut micro-flora, particularly Bacteroides spp. and Eubacterium spp., may contribute to the metabolism of polyphenols, especially flavonoids [90]. Furthermore, anthocyanins and their metabolites regulate the intestinal tract by improving *Lactobacillus* spp., *Enterococcus* spp., *Bifidobacterium* spp. in the poultry gut. Anthocyanins suppress the growth of pathogenic bacteria and improve the organ functionality of poultry and may potentially ameliorate against heat stress [23].

Flavonoids, including those found in cinnamon, act as anti-microbial agents in the poultry gut [23,91]. The composition of cinnamon has also been investigated by other research groups who found that cinnamon bark containing high concentrations of tannins including 3.6% epicatechins and 23.2% PAs [21]. Tannin compounds increase the *Firmicutes* and *Bacteroidetes* in the cecum of broilers and support the growth of *Lactobacillus* by inducing the iron-poor environments in the gut as *Lactobacillus* bacteria do not need iron for their full proliferation and growth. In addition, tannin compounds reduce the *Bacteroides* by decreasing the production of acetate and propionate in the intestinal tract of chickens. The production of SCFAs in the poultry gut is associated with tannic acid [23,41]. Anderson, et al. [92] reported that condensed and hydrolyzable tannin-rich extracts have strong anti-microbial activity against *Campylobacter jejuni*. The ethanolic extract of cinnamon has strong anti-microbial activity against *Salmonella aureus* strains as reported by Bonilla and Sobral [93]. Overall, the effect of cinnamon on gut microbiota of poultry birds given in Table 1.

### 3.6. Effect of Cinnamon on the Immune System

The poultry gut immune system consists of a healthy mucosal layer, intestinal epithelial cells (tightly interconnected), anti-microbial peptides (AMPs) and secreted soluble IgA. The outer loose mucosal layer acts to colonize the bacteria while the compact inner layer repels many bacteria. The mucus layer (the first line of defense against infection) prevents the intestinal microorganisms from penetrating the intestinal epithelium [94]. Another single layer of epithelial cells below the mucus layer separates the environmentally exposed densely colonized intestinal lumen from the subepithelial tissue inside [2,95]. Dietary cinnamon can play a critical role in the prevention/protection of mucus layers from the colonization/invasion of pathogens. Additionally, it can stimulate the gene expression of MUC2 which is a component of the mucus barrier to protect the intestinal epithelial cells. In the absence of MUC2 mucin, pathogenic bacteria easily penetrate into the crypts through epithelial cells and cause severe inflammation [96,97,98]. The exact mechanism of action of cinnamon is not yet elucidated. It is possible that it includes the production of anti-microbial substances, enhancement of the epithelial barrier with the increasing adhesion to the intestinal mucosa, inhibition of pathogens adhesion, and competitive exclusion of pathogens, resulting in the modulation of the immune system. Bacteria such as *Lactobacillus casei* that are increased in poultry consuming cinnamon or cinnamon compounds promote mucus secretions which aid in the barrier function and exclusion of the pathogenic microorganisms.

**Figure 2:**
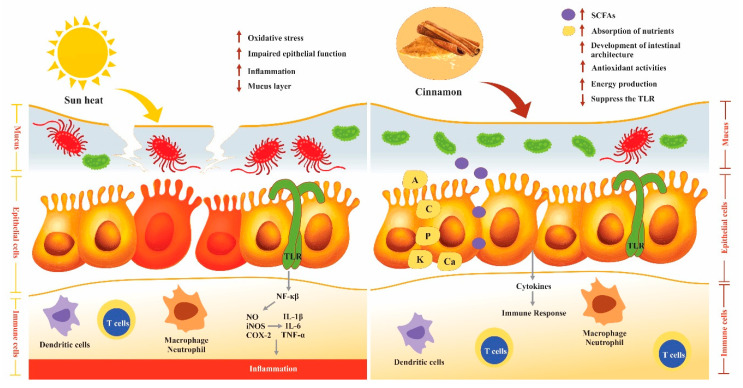
Effect of heat stress on immune response and cinnamon contribution to modulate oxidative stress due to pathogenic bacteria impaired the epithelial cells and mucus and cause inflammation by triggering the toll-like receptors (TLR) (**Left side**). Cinnamon improved the intestinal lumen by improving the growth of commensal bacteria and suppressing the TLRs. Good bacteria produce SCFAs, develop the intestinal architecture and produce energy (**Right Side**).

The maintenance of the gut defensive system is complex and mainly depends on the balance among commensal microflora, mucus and feed. Cinnamon contains phenolic compounds which can scavenge the free radicals and play a significant role in protecting against tissue damage and inflammation [49,64,99]. Dietary cinnamon significantly improved the commensal microbiota, which helps to exclude pathogens, immune stimulation and release nutrients (SCFAs, vitamins and amino acids). The SCFAs act as bacteriostatic agents to eliminate pathogenic bacteria (*Salmonella* spp.) and increase the gastrointestinal absorption surface by the proliferation of gut epithelial cells. Moreover, the production of SCFAs inhibits the conversion of bile into secondary bile products by lowering the pH of the colon. It has been demonstrated through in-vitro studies that cinnamaldehyde inhibited the expression of pro-inflammatory cytokines (IL-1β, IL-6 and TNF-α) in LPS activated J774A.1 cells as well as NO, iNOS and COX-2 expression. Moreover, it also decreased the mRNA expression of chemokines (MIP-1α and MCP-1), which became the cause of inflammation in epithelial cells while increasing anti-inflammatory cytokine IL-10. These studies demonstrated that cinnamaldehyde has anti-inflammatory potential that ultimately improved the immune system in poultry [100,101]. Cinnamaldehyde inhibited the expression of the NF-κβ and interferon regulatory factor-3 (IRF-3) [102]. Furthermore, cinnamaldehyde has immunomodulatory effects related to antigen presentation and humoral immune response [103]. Furthermore, cinnamon reduced the H/L ratio, which is an index of oxidative stress in poultry. To sum up, the use of cinnamon and its bioactive compounds as feed additives in poultry diets have potent effects on antioxidant status, immunity, nutrients availability and digestibility, enzymes secretion, mucus production, gut microbiota and overall poultry health, growth performance and productivity (Figure 2).

## 4. Future Perspective

Economical, safe and healthier poultry production can be achieved by using these PFAs without compromising animal health and growth performance. Nevertheless, the fundamental relationship of these PFAs with increased intestinal microflora will remain a subject matter in the future for elucidative research. The severity of various environmental conditions has a significant impact on poultry health and overall production and CNO may prove beneficial during environmental stress such as heat stress. The immunomodulatory properties of CNO in poultry production have gained more interest and needs more exhaustive research in this field. The corresponding methods should be established for analytical quantification of the bioactive compounds of cinnamon to track the metabolites and their fate in the poultry gut. Concrete scientific knowledge about the biological actions of cinnamon and their compounds will make the application of cinnamon or CNO more successful in poultry industry. This review mainly illustrates the positive effects of cinnamon on the gut microbiota and overall poultry health. More consideration should be given to the chemical composition of cinnamon and its impact on the animal health and productivity.

## 5. Conclusions

To conclude, the inclusion of cinnamon in poultry feed as a PFA has beneficial effects on nutrient digestibility, hypocholesterolaemic, blood biochemical profile, gene expression, immunity, and particularly on gut health to alleviate the impact of disease and environmental stressors. It is suggested that cinnamon supplementation in poultry feed alleviate the stress response by suppressing the NF-κβ pathway and increasing anti-inflammatory cytokines’ expression. This review demonstrates that cinnamon can be used as a PFA in the poultry industry for greater food safety, health and economic aspects.

## Figures and Tables

**Table 1:** Effect of cinnamon on gut microbiota of poultry birds.

Feed Composition	Bird Type	Feed Level	Gut Microbiota	Gut Part	Ref.
* Cinnamon powder	Broiler	10% cinnamon	Total counts of *Enterococcus* spp. and *Lactobacillus* spp. ↑ *Campylobacter* spp. and *E. coli* ↓	Ileum and cecum	[1]
CNO	Broiler	100 mg/kg	*Lactobacillus* and *Bifidobacterium* ↑ *E. coli* ↓	Cecum	[4]
CNO	Broiler	300 mg/kg	No change in *Lactobacillus* spp.; *E. coli* and *Clostridium* spp. ↓	Cecum	[37]
CNO	Japanese quail	200 mg/kg	*Lactobacillus* ↑ *Coliforms* count ↓	intestine	[50]
* CNO	Broiler	500 mg/kg	No effect on total bacterial counts, *E. coli* and *Lactobacillus; Clostridium and Salmonella counts* ↓	Ileum and cecum	[74]
* Cinnamaldehyde	Ross broiler	5 g + 15 g/tonne	*E. coli* ↓	Cecum	[75]
* Cinnamaldehyde	Hubbard broiler	100 mg/kg	*Lactobacillus* counts ↑, *E. coli* and *Clostridium perfringens*↓	Ileum and cecum	[77]

* Combined with other spices. ↑, ↓ represents the increase and decrease of particular bacteria, respectively.

## Data Availability

Not applicable.
